# Gait and turning characteristics from daily life increase ability to predict future falls in people with Parkinson's disease

**DOI:** 10.3389/fneur.2023.1096401

**Published:** 2023-02-28

**Authors:** Vrutangkumar V. Shah, Adam Jagodinsky, James McNames, Patricia Carlson-Kuhta, John G. Nutt, Mahmoud El-Gohary, Kristen Sowalsky, Graham Harker, Martina Mancini, Fay B. Horak

**Affiliations:** ^1^Department of Neurology, Oregon Health & Science University, Portland, OR, United States; ^2^APDM Wearable Technologies, A Clario Company, Portland, OR, United States; ^3^Department of Electrical and Computer Engineering, Portland State University, Portland, OR, United States

**Keywords:** Parkinson's disease, daily life, gait, future falls, turning, inertial sensors

## Abstract

**Objectives:**

To investigate if digital measures of gait (walking and turning) collected passively over a week of daily activities in people with Parkinson's disease (PD) increases the discriminative ability to predict future falls compared to fall history alone.

**Methods:**

We recruited 34 individuals with PD (17 with history of falls and 17 non-fallers), age: 68 ± 6 years, MDS-UPDRS III ON: 31 ± 9. Participants were classified as fallers (at least one fall) or non-fallers based on self-reported falls in past 6 months. Eighty digital measures of gait were derived from 3 inertial sensors (Opal^®^ V2 System) placed on the feet and lower back for a week of passive gait monitoring. Logistic regression employing a “best subsets selection strategy” was used to find combinations of measures that discriminated future fallers from non-fallers, and the Area Under Curve (AUC). Participants were followed *via* email every 2 weeks over the year after the study for self-reported falls.

**Results:**

Twenty-five subjects reported falls in the follow-up year. Quantity of gait and turning measures (e.g., number of gait bouts and turns per hour) were similar in future fallers and non-fallers. The AUC to discriminate future fallers from non-fallers using fall history alone was 0.77 (95% CI: [0.50–1.00]). In contrast, the highest AUC for gait and turning digital measures with 4 combinations was 0.94 [0.84–1.00]. From the top 10 models (all AUCs>0.90) via the best subsets strategy, the most consistently selected measures were variability of toe-out angle of the foot (9 out of 10), pitch angle of the foot during mid-swing (8 out of 10), and peak turn velocity (7 out of 10).

**Conclusions:**

These findings highlight the importance of considering precise digital measures, captured *via* sensors strategically placed on the feet and low back, to quantify several different aspects of gait (walking and turning) during daily life to improve the classification of future fallers in PD.

## Introduction

Falls are prevalent in people with Parkinson's Disease (PD), occurring in 60.5% of patients per year (1). Moreover, fall history is a prominent risk factor for recurring falls in PD, with 39% of patients experiencing recurring falls (1). In addition, people with PD who experience impairments in gait and turning difficulties are significantly more likely to fall at home compared to those with tremor as the primary symptoms (2).

Given the heightened risk of falls in people with PD, it is important to consider which aspects of gait and turning difficulties contribute to fall events. Traditionally, clinical assessments such as the Unified Parkinson's Disease Rating Scale (UPDRS) are used to assess disease severity, gait and turning difficulties (3, 4). Additionally, fall history, as recorded by diary or other self-reports are implemented to monitor falls. Such outcomes, among others, have been shown to be predictors of recurring falls in people with PD (1).

A critical shortcoming of these assessments is the inability to objectively measure gait and turning abnormalities in the patient's everyday environment where falls occur. Furthermore, brief clinical or laboratory assessments of gait and turning (e.g., straight-ahead gait and turning) may not accurately reflect functional mobility of patients in their everyday lives, or capture inherent day-to-day variations in movement patterns that may be indicative of functional capacity (5). Therefore, daily life monitoring of gait and turning could help assess the risk of falling in people with PD, and provide insight into patient behavior outside of traditional testing facilities.

Wearable sensors and advanced algorithms allow researchers to capture objective mobility measures both in the clinic and at home (6–10) that may improve our understanding of fall risk in people with PD. It has been shown that environments and tasks associated with daily living can amplify gait and turning impairments in people with PD (7–9). Given the ubiquity of falls and fall recurrence in PD, researchers have worked to identify digital biomarkers, captured during daily life walking and turning, that can assist with identifying fall risk and optimize clinical trial conduct. Several gait and turning metrics obtained from wearable sensors in laboratory settings and during daily life have been shown to discriminate between fallers and non-fallers in PD (11–15). However, it is still unclear which measures, among the high volume of gait and turning measures calculated from inertial sensors, most accurately discriminate fallers from non-fallers with PD during daily life.

The aim of this study was to investigate if digital measures from different components of gait and turning collected from a week of daily activities increased discriminative ability to predict future falls compared to fall history alone. We hypothesize that variability and turn metrics will best discriminate fallers from non-fallers during daily life, and will show an increased discriminative ability to predict future fallers compared to falls history alone. The main contribution of this study is to show the importance of considering precise digital measures, captured via sensors strategically placed on the feet and low back, to quantify several different aspects of gait (walking and turning) during daily life to improve the classification of future fallers in PD.

## Methods

### Participants

Thirty-four people with idiopathic PD participated in the study. Inclusion criteria were a diagnosis of idiopathic Parkinson's disease from movement disorders specialist with the United Kingdom Parkinson's disease Society Brain Bank criteria, Hoehn & Yahr scale of II-IV, and complaints about gait and balance. Exclusion criteria were the inability to follow protocol instructions, and other factors affecting gait and balance such as musculoskeletal disorders, uncorrected vision or vestibular problems, or inability to stand or walk in the home without an assistive device. The experimental protocol was approved by the Institutional Review Board of the Oregon Health & Science University (eIRB #15578). All the participants provided informed written consent. The same participants have been used in our previous research work comparing gait and turning measures in two levodopa states in the clinic (On vs. Off), and daily life settings (16).

### Clinical assessment

Clinical characteristics (including demographic, motor and cognitive status, and patient-reported outcomes) were assessed with a comprehensive battery of validated tests. Specifically, we collected age, sex, height, weight, disease duration, medications, and the Movement Disorders Society (MDS-revised) Unified Parkinson's disease Rating Scale (MDS-UPDRS) (3); the Hoehn and Yahr Rating Scale; the New Freezing of Gait Questionnaire (NFoGQ) (17); the Parkinson's Disease Questionnaire-39 (PDQ-39); and the Montreal Cognitive Assessment (MoCA) (18).

### Falls data collection

Self-reported fall history based on the previous 6 months was collected and participants were classified as fallers (at least one fall) or non-fallers based on falls history prior to the study visit. For future falls, following a week of continuous monitoring of gait. participants were asked complete a 12-month, fall-monitoring period immediately after the 1 week of daily life gait data collection. Participants received bimonthly emails to indicate if they experienced a fall or near fall during the previous 2 weeks. If participants failed to respond, a research assistant called them to ascertain if they had fallen in the previous 2 weeks. A fall was defined as “an event that results in coming to rest unintentionally on the ground or other lower level”. Future-fallers were classified as participants with >1 fall in the 12-month period after daily life gait data collection. If a fall(s) occurred, we collected number of falls and nature of injury.

### Daily life data collection

Participants were asked to wear 2 Opal-instrumented socks, one on each foot, and an Opal sensor over the lower lumbar area with an elastic belt (APDM Wearable Technologies-a Clario Company, Portland, OR, USA) for a week of continuous monitoring of at least 8 hours/day during daily activities including both on and off states. The details of the instrumented socks were previously described in Shah et al. (9). Briefly, instrumented socks incorporated the same inertial sensors on top of the foot as used in the Opal, with the battery separated from the sensor and positioned just above the lateral malleolus. Each Opal sensor includes tri-axial accelerometer, gyroscope, and magnetometer and was configured to sample at a rate of 128 Hz. The Opal is lightweight (22 g), has a battery life of 12 h, and includes 8 GB of storage, which can record over 30 days of data.

Participants were asked to remove the sensors at night and plugged in to recharge the batteries. During the daily activities, data were continuously collected and stored in the internal memory of the Opals. Participants were asked to mail back the sensors using a pre-paid mailing box after completion of a week of data collection. Once we receive the devices, the raw data were uploaded to a secure cloud-based database on Amazon Web Server (AWS), processed on the same server and calculated gait metrics were then downloaded to a local computer for further analysis.

### Digital gait and turning measures during daily life

The algorithms used to calculate the measures of gait and turning were the same for the laboratory and daily life data as were detailed previously (19). In summary, the daily life algorithm first searches for possible bouts of walking from inertial sensor data from the feet using a time-domain approach. Second, individual steps are combined into potential bouts of walking if the duration from one step to the next step is less than 2.5 seconds. Finally, each possible bout that contains at least 3 seconds in duration and at least 3 steps is processed with the commercial gait analysis algorithms included in Mobility Lab V2 for prescribed gait tests (APDM Wearable Technologies, A Clario company) (20). For the gait measures reported in this paper, we calculated a mean and variability across all strides over the week of recording and included only the periods of straight walking. Straight walking were periods of walking in which the heading angle of the foot during stance changed by no more than 20 degrees during a single stride and that did not contain detected turns as determined from the lumbar sensor (21). For turning measures, we used a previously published algorithm to detect and characterize each turn (21). Specifically, all the turns with an amplitude larger than 40 degrees were detected as a turn and we did not restrict any particular range of turns but considered all. In total, we derived 52 measures and grouped into four domains (Lower Body, Lower Trunk, Turning, and Variability) similar to described in Shah et al. (22).

### Statistical analysis

The normality of data was examined by the Shapiro-Wilk test. For the demographics measures that were non-normally distributed, the Mann- Whitney U test was used to compare fallers and non-fallers. Otherwise, independent samples *t*-test (or Chi-squared test) was used to examine possible group differences.

To investigate which combination of digital gait and turning measures discriminate fallers from non-fallers group, we used logistic regression employing a best subset selection (23). The best subset selection strategy selects the best model from all possible subsets according to goodness-of-fit criteria. To assess the goodness-of-fit, we used the Bayesian Information Criteria (BIC) (23). We selected the top 15 models based on BIC for two, three, and finally for four digital outcome measures of mobility (15^*^3 = 45 models total). Finally, we computed the Area Under the ROC Curve (AUC) using “ROC” function (empirical ROC) in R (24, 25) and ranked the top 10 models based on the AUC. All statistical analysis was performed using R Version 1.1.456 software.

### Power analysis

We recently showed that variability of the number of steps during turning was a sensitive metric in predicting falls in the 6 months after the week of continuous monitoring in a group of healthy elderly fallers (26). Out of 35 healthy elderly participants (sample of convenience), 7 fell at least once in the 6 months after the week of continuous monitoring. To determine the number of subjects needed in this study, we compared the variability of the number of steps needed to complete a turn by subjects who experienced one or more falls to variability in the subjects that did not fall. Given the fallers group mean variability of 0.59 (SD 0.04) and the non-fallers group mean of 0.54 (SD 0.03), for alpha = 0.05 and a power of 95%, we are adequately powered to separate fallers and non-fallers with a sample size of 12 subjects per group.

## Results

### Group characteristics and adherence

From a total of 34 people with PD, 17 were fallers and 17 were non-fallers based on self-reported fall history. Table 1 compares the demographic characteristics between non-fallers and fallers. The demographic and other digital measures mostly followed the normal distribution and we did not find any multimodal distribution. There were no significant differences between the groups for demographic characteristics, smart socks compliance and activity measures from daily life. After 1-year follow up from the data collection, out of 34 people, 25 people were fallers and 9 people were non-fallers (see Supplementary Table S1 for number of past falls and future falls for each subject).

**Table 1:** Participant demographic information for non-faller and faller groups.

	**Non-fallers (*N* = 17)**	**Fallers (*N* = 17)**	***p***
Age (yrs)	66.82 (6.61)	68.69 (11.10)	0.29
Disease Duration (yrs)	7.29 (5.6)	9.24 (4.58)	0.14
H and Y ON (#)	2 (0)	2.18 (0.53)	0.164
H and Y OFF (#)	2.06 (0.24)	2.29 (0.59)	0.153
MDS-UPDRS Part III total score ON (#)	29.47 (8.49)	32.65 (9.92)	0.36
MDS-UPDRS Part III total score OFF (#)	43.88 (11.3)	46.18 (10.02)	0.39
MDS-UPDRS Part III PIGD score ON (#)	2.59 (1.42)	3.53 (2.62)	0.34
MDS-UPDRS Part III PIGD score OFF (#)	3.53 (1.66)	5.35 (3.28)	0.09
MoCA total score (#)	26.94 (2.38)	26.88 (2.93)	0.81
LEDD total score (mg/day)	1,541.94 (2,342.53)	1,128.1 (533.18)	0.36
PDQ39 total score (%)	13.91 (7.3)	23.3 (14.82)	0.13
PDQ39 Mobility score (%)	11.91 (12.14)	21.76 (18.68)	0.11
MDS-UPDRS Dyskinesia ON (#)	0.35 (0.49)	0.53 (0.51)	0.31
NFOGQ past month (#)	0.47 (0.51)	0.76 (0.44)	0.08
**Activity measures from daily life**			
Number of days	6.76 (0.56)	6.41 (1.28)	0.31
Total hours of recording	64.57 (8.64)	62.07 (16.02)	0.58
Bouts/hours (#)	7.82 (3.05)	7.65 (4.16)	0.70
Strides/hours (#)	149.87 (60.95)	161.07 (94.82)	0.85
Turns/hours (#)	20.19 (9.33)	21.34 (15.71)	0.97

H and Y, Hoehn and Yahr scale; MDS-UPDRS Part III, Movement Disorders Society-Unified Parkinson's Disease Rating Scale, motor sub-score; MoCA, Montreal Cognitive Assessment; LEDD, levodopa equivalent daily dose; PDQ39, Parkinson's Disease Questionnaire-39; NFOGQ Past Month, First question of NFOGQ for freezing in last month.

### Digital gait and turning measures separating fallers from non-fallers during daily life

**Figure 1:**
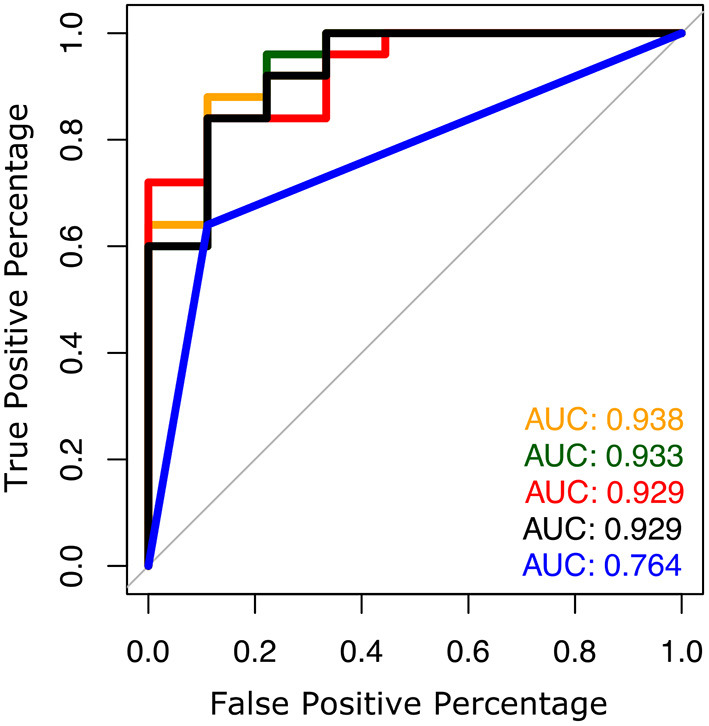
ROC plots to predict future falls based on falls history alone (blue line) and various combinations of gait measures (top 4 from Table 2).

**Figure 2:**
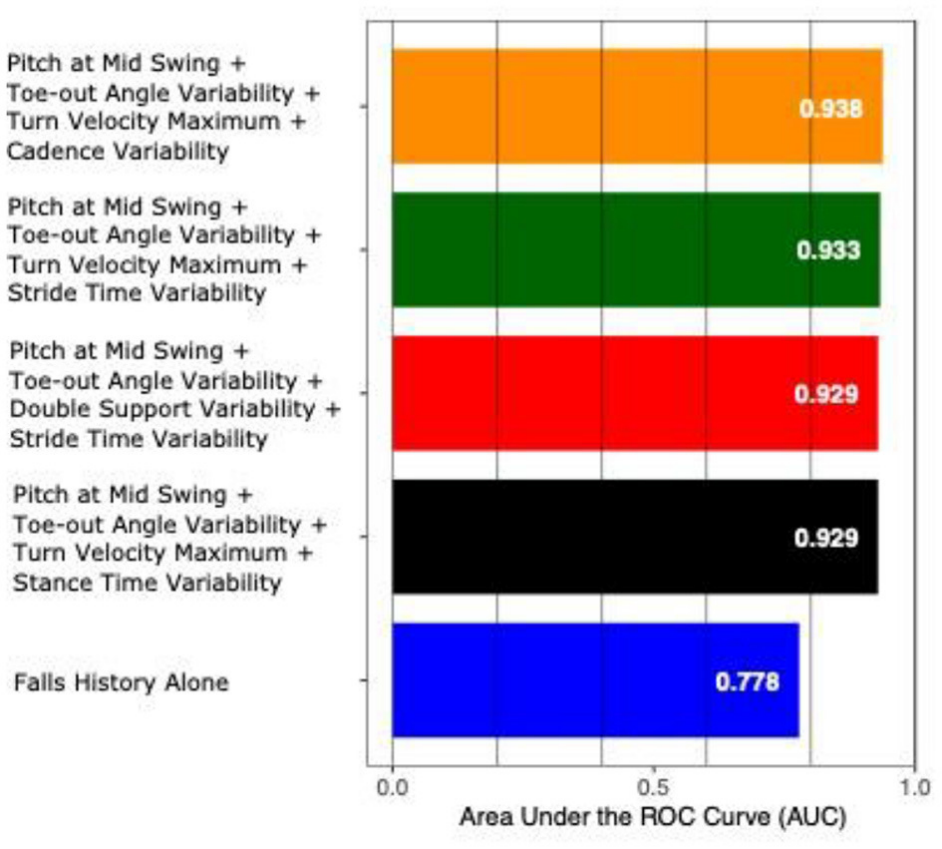
AUC values to predict future falls based on falls history alone and various combinations of gait measures (top 4 from Table 2).

The AUC to discriminate future fallers from non-fallers using fall history alone was 0.77 (95% CI: [0.50–1.00]). In contrast, the highest AUC for gait and turning digital measures with 4 combinations was 0.94 [0.84–1.00]. From the top 10 models (all AUCs > 0.90) *via* the best subsets strategy, the most consistently selected gait measures were variability of toe-out angle of foot (9x), pitch angle of the foot during mid-swing (8x), and the maximum average turn velocity (7x) (see Table 2). Figures 1, 2 show the ROC curves and AUC values for the top 4 fall prediction models selected *via* best subsets of gait metrics strategy and using falls history alone.

**Table 2:** Combination of digital gait measures that best discriminated future fallers from non-fallers in PD during daily life.

**Digital measures of gait and turning**								**AUC**
**1st**		**2nd**		**3rd**		**4th**		
	Pitch angle of the foot during mid-swing		Toe-out angle variability		Turn velocity maximum		Cadence variability	0.94 (0.84–1)
	Pitch angle of the foot during mid-swing		Toe-out angle variability		Turn velocity Maximum		Stride time variability	0.93 (0.82–1)
	Pitch angle of the foot during mid-swing		Toe-out angle variability		Double support variability		Stride time variability	0.93 (0.83–0.99)
	Pitch angle of the foot during mid-swing		Toe-out angle variability		Turn velocity maximum		Stance time variability	0.93 (0.82–1)
	Pitch angle of the foot during mid-swing		Turn angle		Turn velocity maximum		Stride time variability	0.93 (0.82–1)
	Pitch angle of the foot during mid-swing		Toe-out angle variability		Turn velocity maximum		–	0.92 (0.81–1)
	Pitch angle of the foot during mid-swing		Toe-out angle variability		Double support variability		Stance time variability	0.92 (0.81–0.99)
	Pitch angle of the foot at toe-off		Toe-out angle variability		Turn velocity maximum		Cadence variability	0.91 (0.79–1)
	Pitch angle of the foot maximum at toe-off		Toe-out angle variability		Turn velocity maximum		Stride time variability	0.91 (0.78–1)
	Pitch angle of the foot during mid-swing		Toe-out angle variability		Trunk transverse range of motion		Stride time variability	0.91 (0.78–0.99)

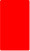
 Lower body. 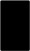 Variability. 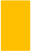 Turning. 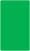 Lower trunk.

Considering the definition of the recurrent fallers (*n* = 20 fallers and 14 non-fallers), the most consistently selected gait measures were the pitch angle of the foot during mid-swing (8x), stride time variability (8x), and foot-strike angle variability (7x) (see Supplementary Table S2).

## Discussion

This study offers preliminary evidence that different aspects of gait and turning during daily life (specifically, gait, turning, and variability domains) are important to predict future fallers. Further, digital measures from different components of gait showed more discriminative ability to predict future fallers from non-fallers compared to falls history, alone.

The top ten models incorporating digital gait and turning measures in this study were able to separate fallers from non-fallers with an AUC over 0.90 compared to fall history alone, which yielded an AUC of 0.77. Gait variability was the most consistent domain selected, with toe out angle variability being the most common variability measure selected, followed by stride time variability. These findings are consistent with previous research showing association between gait variability and fall risk in people with PD (27–29).

Digital measures of gait and turning have been shown to have a good predictive value for a fall risk. For an example, Van Schooten et al. reported an AUC of 0.82 when assessing predictive value of accelerometry based measures of gait for detecting falls in 169 older adults (30). In 26 patients with multiple sclerosis, toe-off angle in daily living has been identified as a significant predictor of falls in patients with multiple sclerosis, with an AUC of 0.86 (31). Additionally, an AUC of 0.93 was reported using clinical and functional characteristics in a multivariate model of fall prediction in 49 patients with PD (22), while fall classification accuracies of between 70–80% have been reported using machine learning models with gait metrics as principal predictors in 251 patients with PD (13). In this study, gait and turning domains were most consistently selected by best subset selection following the variability domain. Specifically, pitch angle (dorsiflexion) of the foot during mid-swing and peak turn velocity were most prevalent. Pitch angle of the foot is a particularly pertinent measure for fall risk as it reflects the amount of toe clearance achieved by the participant during mid swing, and thus may contribute to trips or stumbles while walking. Additionally, daily life turning characteristics in people with PD have been shown to be significantly impaired compared to age-matched controls (9, 21, 32–34), and turning is associated with falls in older adults (35, 36).

Findings from several recent studies highlight gait, variability, and turning domains in daily life as particularly relevant to understanding PD disease severity and fall prediction during daily living. del Din et al. showed that daily-living gait and variability measures, collected with a wearable sensor, were significantly different in individuals with PD compared to controls (7), and thereafter showed that similar domains were significantly different between fallers with PD compared to non-fallers with PD (11). Galperin et al. used a wearable sensor for 7 days of daily-living monitoring of individuals with PD and a history of falls (37). Their findings showed that daily-living gait and variability measures accounted for 62% of explained variance in the MDS-UPDRS- part III scores of fallers with PD, followed by laboratory measures (30%) and participant demographics/characteristics (7%) (37). More recently, Shah et al. demonstrated that gait, turning, and variability measures, captured with wearable sensors during 1 week of continuous home monitoring, were most significant in distinguishing patients with PD from healthy controls (19). Our results suggest that turning might be more important in identifying the patients who are at risk of their first fall, while gait variability might be more important in identifying the recurrent fallers.

These findings provide support for the collection of digital gait, variability, and turning markers to objectively assess fall risk of people with PD during daily life. Notably, three body worn sensors were required to capture gait, turning, and variability domains during daily life monitoring. The use of instrumented socks to capture mobility of each foot represents a novel approach that may be useful for home monitoring during clinical trials, as they are less obtrusive for continuous monitoring compared to sensors strapped to the foot. Moreover, implementing three body worn sensors allows for more accurate measurement of gait, variability, and turning domains compared to a single lumbar sensor, which neglects to capture foot angle and variability of foot placement.

There are several limitations of the current study. First, we recommend caution in interpreting the results as the individual models from this small size study are not yet validated in a separate cohort. Hence the performance of the models may be optimistic. Second, future studies with larger cohorts are needed to validate these preliminary findings. Third, we performed the analysis by taking the mean of each measure for all the strides over a week for each subject and thus gave equal weight to each stride. But in reality, gait speed and other measures vary for gait bouts of different lengths (7, 38–40). Hence, future work will focus on how gait bout length affects the discriminatory power of the proposed fall models. Forth, the follow-up period may also affect the ability of fall history to predict future falls. Finally, test-retest reliability and sensitivity of the top measures related to disease progression and falls should be investigated to explore the utility of these digital endpoints for clinical trials.

## Conclusion

Inertial sensors worn on the feet and lumbar level for 7 days provided measures of gait pace, variability and turning that increased the ability to predict future falls in people with PD, beyond predictions from fall history alone.

## Data availability statement

The raw data supporting the conclusions of this article will be made available by the authors, without undue reservation.

## Ethics statement

This study involved human participants; the protocol was reviewed and approved by the Institutional Review Board of Oregon Health & Science University (eRIB #15578). All participants provided written informed consent to participate in this study.

## Author contributions

VS: conception, organization, execution of research project, design, execution, review and critique of statistical analysis, writing of the first draft, and review and critique of manuscript preparation. AJ: writing of the first draft and review and critique of manuscript preparation. JM, JN, ME-G, and KS: review and critique of the statistical analysis and manuscript preparation. PC-K: organization and execution of research project, review and critique of statistical analysis, and review and critique of manuscript preparation. GH: organization and execution of research project and review and critique of manuscript preparation. MM and FH: conception and organization of research project, design, review and critique of statistical analysis, and review and critique of manuscript preparation. ME-G and JM: conception. All authors contributed to the article and approved the submitted version.
