# Supplementary material for: Gait and turning characteristics from daily life increase ability to predict future falls in people with Parkinson's disease
Source: Front Neurol. 2023 Feb 28;14:1096401. doi: 10.3389/fneur.2023.1096401 (PMC10015637; doi:10.3389/fneur.2023.1096401)
Supplement: Supplementary file 1 [file Data_Sheet_1.docx]

**Gait and Turning Characteristics from Daily Life Increase Ability to Predict Future Falls**

**in People with Parkinson’s Disease**

Supplementary Table S1: Number of past and future falls for each subject.

| SubID | Past 6 months falls | Future 12 months falls |
| --- | --- | --- |
| PD_01 | 0 | 1 |
| PD_02 | 0 | 0 |
| PD_03 | 2 | 2 |
| PD_04 | 2 | 9 |
| PD_05 | 0 | 0 |
| PD_06 | 1 | 3 |
| PD_07 | 0 | 2 |
| PD_08 | 0 | 2 |
| PD_09 | 0 | 2 |
| PD_10 | 1 | 5 |
| PD_11 | 0 | 0 |
| PD_12 | 60 | 27 |
| PD_13 | 2 | 61 |
| PD_14 | 3 | 9 |
| PD_15 | 0 | 0 |
| PD_16 | 10 | 8 |
| PD_17 | 4 | 5 |
| PD_18 | 5 | 6 |
| PD_19 | 0 | 1 |
| PD_20 | 2 | 4 |
| PD_21 | 0 | 2 |
| PD_22 | 0 | 0 |
| PD_23 | 0 | 0 |
| PD_24 | 1 | 1 |
| PD_25 | 0 | 1 |
| PD_26 | 0 | 0 |
| PD_27 | 0 | 0 |
| PD_28 | 1 | 6 |
| PD_29 | 1 | 2 |
| PD_30 | 1 | 0 |
| PD_31 | 0 | 4 |
| PD_32 | 3 | 2 |
| PD_33 | 0 | 1 |
| PD_34 | 2 | 2 |

Supplementary Table S2: Combination of digital gait measures that best discriminate future recurrent fallers from non-fallers in PD during daily life.

| **Digital measures of gait and turning** | | | | | | | | AUC |
| --- | --- | --- | --- | --- | --- | --- | --- | --- |
| 1st | | 2nd | | 3rd | | 4th | |  |
|  | Pitch angle of the foot  during mid-swing |  | Toe-out angle  variability |  | Foot-strike angle variability |  | Cadence  variability | 0.91 (0.80-98) |
|  | Pitch angle of the foot  during mid-swing |  | Toe-out angle  variability |  | Foot-strike angle variability |  | Stride time  variability | 0.91 (0.79-99) |
|  | Pitch angle of the foot  during mid-swing |  | Turn angle |  | Foot-strike angle variability |  | Stride time  variability | 0.90 (0.78-0.99) |
|  | Pitch angle of the foot  during mid-swing |  | Turn velocity  Maximum variability |  | Foot-strike angle variability |  | Stride time  variability | 0.89 (0.76-0.99) |
|  | Pitch angle of the foot  during initial contact |  | Toe-out angle  variability |  | Stance time  variability |  | Stride time  variability | 0.89 (0.76-0.99) |
|  | Pitch angle of the foot  during mid-swing |  | Toe-out angle  variability |  | Foot-strike angle variability |  | Swing time variability | 0.88 (0.74-0.97) |
|  | Elevation at mid-swing |  | Toe-out angle  variability |  | Stride length  variability |  | Cadence  variability | 0.88 (0.74-0.98) |
|  | Pitch angle of the foot  during mid-swing |  | Toe-out angle  variability |  | Stride length  variability |  | Stride time  variability | 0.88 (0.74-0.97) |
|  | Pitch angle of the foot  during mid-swing |  | Pitch angle of the foot at toe-off |  | Foot-strike angle variability |  | Stride time  variability | 0.88 (0.74-0.97) |
|  | Pitch angle of the foot  during mid-swing |  | Pitch angle of the foot maximum at toe-off |  | Foot-strike angle variability |  | Stride time  variability | 0.88 (0.74-0.97) |
